# Ethyl 2-amino-4-(4-fluoro­phen­yl)-6-meth­oxy-4*H*-benzo[*h*]chromene-3-carboxyl­ate

**DOI:** 10.1107/S1600536812021939

**Published:** 2012-05-19

**Authors:** Ahmed M. El-Agrody, Mohamed A. Al-Omar, Abdel-Galil E. Amr, Tze Shyang Chia, Hoong-Kun Fun

**Affiliations:** a Chemistry Department, Faculty of Science, King Khalid University, 9004, Abha, Saudi Arabia; b Pharmaceutical Chemistry Department, College of Pharmacy, King Saud University, Riyadh 11451, Saudi Arabia; c Drug Exploration & Development Chair (DEDC), College of Pharmacy, King Saud University, Riyadh 11451, Saudi Arabia; d Applied Organic Chemistry Department, National Research Center, Dokki 12622, Cairo, Egypt; e X-ray Crystallography Unit, School of Physics, Universiti Sains Malaysia, 11800 USM, Penang, Malaysia

## Abstract

In the title compound, C_23_H_20_FNO_4_, the fluoro-substituted benzene ring is approximately perpendicular to the mean plane of the 4*H*-benzo[*h*]chromene ring system [maximum deviation = 0.264 (1) Å], with a dihedral angle of 83.79 (6)°. The pyran ring adopts a flattened boat conformation. The meth­oxy group is slightly twisted from the attached benzene ring of the 4*H*-benzo[*h*]chromene moiety [C—O—C—C = −2.1 (2)°]. An intra­molecular N—H⋯O hydrogen bond generates an *S*(6) ring motif. In the crystal, mol­ecules are linked by N—H⋯O and N—H⋯F hydrogen bonds into a layer parallel to the *bc* plane. The crystal packing also features C—H⋯π inter­actions.

## Related literature

For background to and applications of 4*H*-chromene and its derivatives, see: Jeso & Nicolaou (2009 ▶); Alvey *et al.* (2008 ▶, 2009 ▶); Symeonidis *et al.* (2009 ▶); Brühlmann *et al.* (2001 ▶); Bedair *et al.* (2001 ▶); El-Agrody *et al.* (2002 ▶, 2011 ▶); Abd-El-Aziz *et al.* (2004 ▶); Sabry *et al.* (2011 ▶). For ring puckering parameters, see: Cremer & Pople (1975 ▶). For hydrogen-bond motifs, see: Bernstein *et al.* (1995 ▶).
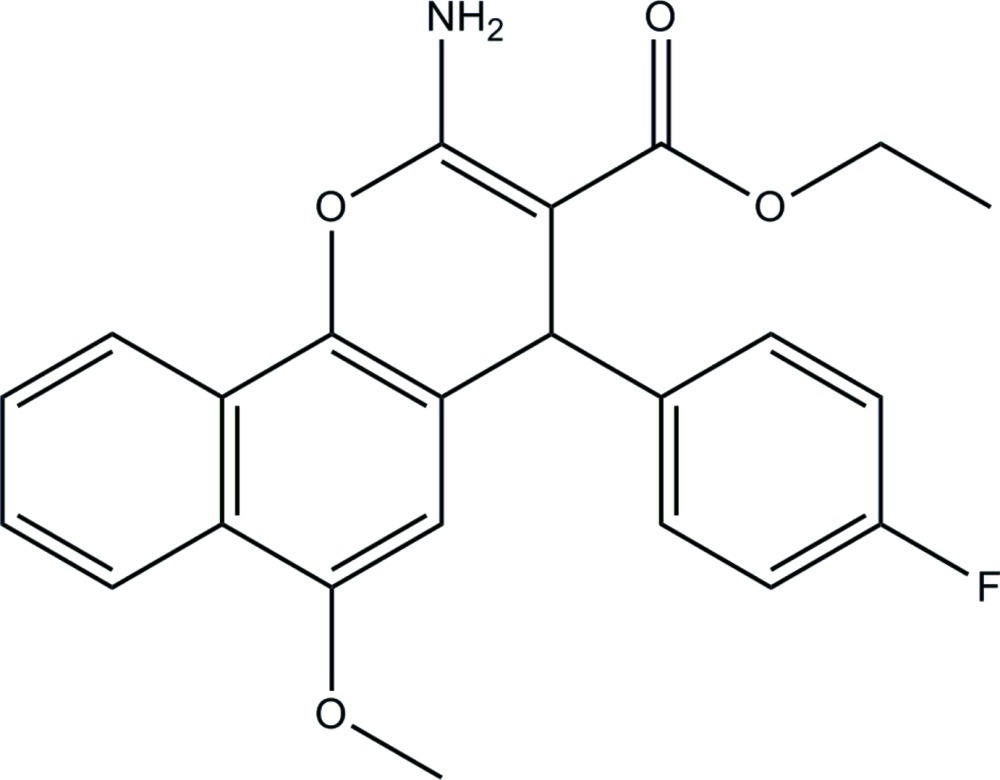


## Experimental

### 

#### Crystal data

C_23_H_20_FNO_4_
*M* *_r_* = 393.40
Monoclinic, 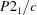

*a* = 12.6844 (3) Å
*b* = 16.1933 (4) Å
*c* = 9.4579 (2) Å
β = 94.288 (2)°
*V* = 1937.24 (8) Å^3^
*Z* = 4
Cu *K*α radiation
μ = 0.82 mm^−1^
*T* = 296 K
0.81 × 0.74 × 0.04 mm

#### Data collection

Bruker SMART APEXII CCD area-detector diffractometer
Absorption correction: multi-scan (*SADABS*; Bruker, 2009 ▶) *T*_min_ = 0.556, *T*_max_ = 0.972
13713 measured reflections
3657 independent reflections
3009 reflections with *I* > 2σ(*I*)
*R*_int_ = 0.034

#### Refinement

*R*[*F* ^2^ > 2σ(*F* ^2^)] = 0.039
*wR*(*F* ^2^) = 0.117
*S* = 1.08
3657 reflections
273 parameters
H atoms treated by a mixture of independent and constrained refinement
Δρ_max_ = 0.19 e Å^−3^
Δρ_min_ = −0.19 e Å^−3^

### 

Data collection: *APEX2* (Bruker, 2009 ▶); cell refinement: *SAINT* (Bruker, 2009 ▶); data reduction: *SAINT*; program(s) used to solve structure: *SHELXTL* (Sheldrick, 2008 ▶); program(s) used to refine structure: *SHELXTL*; molecular graphics: *SHELXTL*; software used to prepare material for publication: *SHELXTL* and *PLATON* (Spek, 2009 ▶).

## Supplementary Material

Crystal structure: contains datablock(s) global, I. DOI: 10.1107/S1600536812021939/is5141sup1.cif

Structure factors: contains datablock(s) I. DOI: 10.1107/S1600536812021939/is5141Isup2.hkl

Supplementary material file. DOI: 10.1107/S1600536812021939/is5141Isup3.cml

Additional supplementary materials:  crystallographic information; 3D view; checkCIF report

## Figures and Tables

**Table 1:** Hydrogen-bond geometry (Å, °) *Cg*1, *Cg*2 and *Cg*3 are the centroids of C4–C6/C11–C13, C14–C19 and C6–C11 rings, respectively.

*D*—H⋯*A*	*D*—H	H⋯*A*	*D*⋯*A*	*D*—H⋯*A*
N1—H2*N*1⋯O3^i^	0.89 (2)	2.23 (2)	3.0969 (19)	165.8 (17)
N1—H1*N*1⋯O3	0.89 (2)	2.111 (18)	2.7570 (18)	129.1 (16)
N1—H1*N*1⋯F1^ii^	0.89 (2)	2.32 (2)	3.034 (2)	137.6 (16)
C8—H8*A*⋯*Cg*1^iii^	0.93	2.81	3.5633 (16)	139
C10—H10*A*⋯*Cg*2^iv^	0.93	2.94	3.7003 (17)	140
C20—H20*C*⋯*Cg*3^iv^	0.96	2.74	3.5896 (17)	148

Symmetry codes: (i) 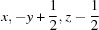; (ii) 
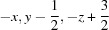
; (iii) 
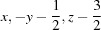
; (iv) 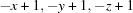.

**Fig. 1.**
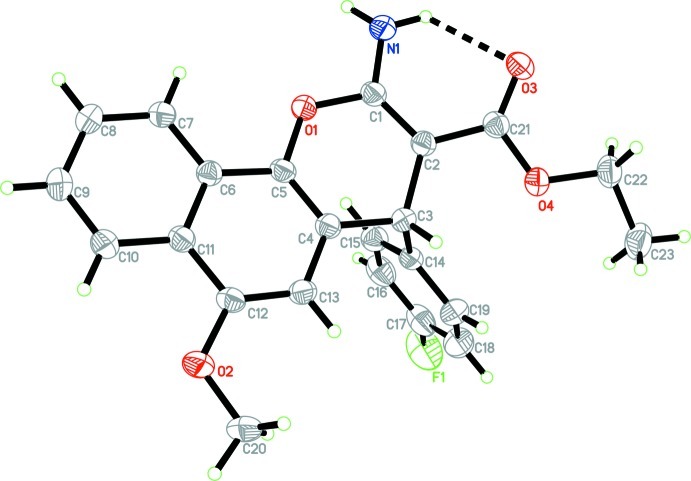
The molecular structure of the title compound with atom labels and 30% probability displacement ellipsoids.

**Fig. 2.**
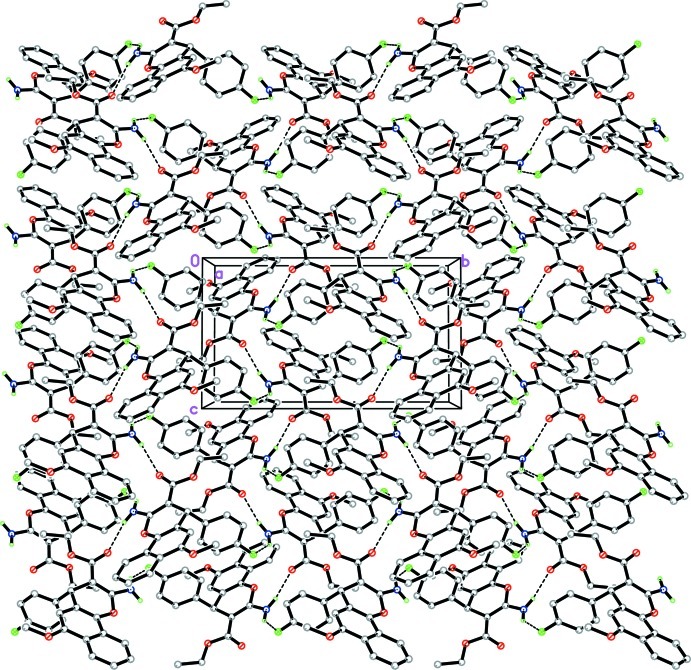
A packing view of the title compound along the a axis. The dashed lines represent the hydrogen bonds. For clarity sake, hydrogen atoms not involved in hydrogen bonding have been omitted.

## supplementary crystallographic information

### Comment

The 4*H*-chromene nucleus is frequently found in bioactive compounds and plays an important role in biochemical processes (Jeso & Nicolaou, 2009; Alvey *et al.*, 2008, 2009; Symeonidis *et al.*, 2009). In addition, 4*H*-chromenes and fused 4*H*-chromenes nuclei are used in treatment of Alzheimer's disease and Schizophrenia disorder (Brühlmann *et al.*, 2001). In view of the above observations and in continuation of our program on the chemistry of 4*H*-pyran derivatives (Bedair *et al.*, 2001; El-Agrody *et al.*, 2002, 2011; Abd-El-Aziz *et al.*, 2004; Sabry *et al.*, 2011), we report herein the crystal structure of the title compound.

The asymmetric unit of the title compound is shown in Fig. 1. The fluoro-substituted benzene ring (C14–C19) is approximately perpendicular to the 4*H*-benzo[*h*]chromene ring system [O1/C1–C13, maximum deviation = 0.264 (1) Å at atom C2] as indicated by the dihedral angle of 83.79 (6)°. The pyran ring (O1/C1–C5) adopts a flattened boat conformation [puckering parameters (Cremer & Pople, 1975), *Q* = 0.2599 (13) Å, θ = 79.3 (3)° and φ = 170.9 (3)°]. The atoms O1 and C3 are deviating from the mean plane of C1/C2/C4/C5 by 0.1589 (18) and 0.2820 (21) Å, respectively. The methoxy group (C20/O2) is slightly twisted from the attached benzene ring (C4–C6/C11–C13) of the 4*H*-benzo[*h*]chromene moiety with the torsion angle C20—O2—C12—C13 of -2.1 (2)°. An intramolecular N1—H1N1···O3 hydrogen bond generates an S(6) ring motif (Bernstein *et al.*, 1995) in the molecule.

In the crystal (Fig. 2), molecules are linked by intermolecular N1—H2N1···O3 and N1—H1N1···F1 hydrogen bonds (Table 1) into a layer parallel to the *bc* plane. The crystal packing is further stabilized by C—H···π interactions (Table 1), involving *Cg*1, *Cg*2 and *Cg*3 which are the centroids of C4–C6/C11–C13, C14–C19 and C6–C11 rings, respectively.

### Experimental

A solution of 4-methoxy-1-naphthol (0.01 mol) in EtOH (30 ml) was treated with ethyl α-cyano-*p*-fluorocinnamate (0.01 mol) and piperidine (0.5 ml). The reaction mixture was heated under reflux for 2 h. The obtained solid product was collected by filtration, dried and crystallized from ethanol to give the title compound. *M.p.*: 435–436 K.

### Refinement

The atoms H1N1 and H2N1 were located in a difference Fourier map and refined freely [N—H = 0.88 (2) and 0.89 (2) Å]. The remaining H atoms were positioned geometrically (C—H = 0.93, 0.96, 0.97 and 0.98 Å) and refined using a riding model with *U*_iso_(H) = 1.2 or 1.5*U*_eq_(C). A rotating group model was applied to the methyl groups.

### Crystal data

**Table:**

C_23_H_20_FNO_4_	*F*(000) = 824
*M**_r_* = 393.40	*D*_x_ = 1.349 Mg m^−^^3^
Monoclinic, *P*2_1_/*c*	Cu *K*α radiation, λ = 1.54178 Å
Hall symbol: -P 2ybc	Cell parameters from 3029 reflections
*a* = 12.6844 (3) Å	θ = 3.5–66.5°
*b* = 16.1933 (4) Å	µ = 0.82 mm^−^^1^
*c* = 9.4579 (2) Å	*T* = 296 K
β = 94.288 (2)°	Plate, colourless
*V* = 1937.24 (8) Å^3^	0.81 × 0.74 × 0.04 mm
*Z* = 4	

### Data collection

**Table:**

Bruker SMART APEXII CCD area-detector diffractometer	3657 independent reflections
Radiation source: fine-focus sealed tube	3009 reflections with *I* > 2σ(*I*)
Graphite monochromator	*R*_int_ = 0.034
φ and ω scans	θ_max_ = 70.1°, θ_min_ = 3.5°
Absorption correction: multi-scan (*SADABS*; Bruker, 2009)	*h* = −15→15
*T*_min_ = 0.556, *T*_max_ = 0.972	*k* = −19→19
13713 measured reflections	*l* = −11→11

### Refinement

**Table:**

Refinement on *F*^2^	Secondary atom site location: difference Fourier map
Least-squares matrix: full	Hydrogen site location: inferred from neighbouring sites
*R*[*F*^2^ > 2σ(*F*^2^)] = 0.039	H atoms treated by a mixture of independent and constrained refinement
*wR*(*F*^2^) = 0.117	*w* = 1/[σ^2^(*F*_o_^2^) + (0.0641*P*)^2^ + 0.2106*P*] where *P* = (*F*_o_^2^ + 2*F*_c_^2^)/3
*S* = 1.08	(Δ/σ)_max_ < 0.001
3657 reflections	Δρ_max_ = 0.19 e Å^−^^3^
273 parameters	Δρ_min_ = −0.19 e Å^−^^3^
0 restraints	Extinction correction: *SHELXTL* (Sheldrick, 2008), Fc^*^=kFc[1+0.001xFc^2^λ^3^/sin(2θ)]^-1/4^
Primary atom site location: structure-invariant direct methods	Extinction coefficient: 0.0059 (5)

### Special details

**Table:**

Geometry. All e.s.d.'s (except the e.s.d. in the dihedral angle between two l.s. planes) are estimated using the full covariance matrix. The cell e.s.d.'s are taken into account individually in the estimation of e.s.d.'s in distances, angles and torsion angles; correlations between e.s.d.'s in cell parameters are only used when they are defined by crystal symmetry. An approximate (isotropic) treatment of cell e.s.d.'s is used for estimating e.s.d.'s involving l.s. planes.
Refinement. Refinement of *F*^2^ against ALL reflections. The weighted *R*-factor *wR* and goodness of fit *S* are based on *F*^2^, conventional *R*-factors *R* are based on *F*, with *F* set to zero for negative *F*^2^. The threshold expression of *F*^2^ > σ(*F*^2^) is used only for calculating *R*-factors(gt) *etc*. and is not relevant to the choice of reflections for refinement. *R*-factors based on *F*^2^ are statistically about twice as large as those based on *F*, and *R*- factors based on ALL data will be even larger.

### Fractional atomic coordinates and isotropic or equivalent isotropic displacement parameters (Å^2^)

**Table:**

	*x*	*y*	*z*	*U*_iso_*/*U*_eq_	
F1	0.04716 (11)	0.70219 (9)	0.55146 (16)	0.1144 (5)	
O1	0.28914 (8)	0.30595 (6)	0.72092 (10)	0.0487 (3)	
O2	0.63332 (8)	0.50557 (7)	0.64293 (13)	0.0584 (3)	
O3	0.10800 (9)	0.34723 (7)	1.07127 (11)	0.0559 (3)	
O4	0.16230 (8)	0.47933 (6)	1.06692 (10)	0.0516 (3)	
N1	0.16357 (11)	0.25396 (8)	0.84457 (17)	0.0554 (3)	
C1	0.22572 (10)	0.31944 (8)	0.82843 (14)	0.0429 (3)	
C2	0.22913 (10)	0.39123 (8)	0.90464 (13)	0.0416 (3)	
C3	0.29340 (10)	0.46421 (8)	0.85933 (14)	0.0413 (3)	
H3A	0.3264	0.4904	0.9450	0.050*	
C4	0.38041 (10)	0.43437 (8)	0.77125 (13)	0.0408 (3)	
C5	0.37413 (10)	0.35926 (8)	0.70758 (14)	0.0404 (3)	
C6	0.45447 (10)	0.32854 (8)	0.62507 (13)	0.0405 (3)	
C7	0.45043 (11)	0.24931 (9)	0.56213 (14)	0.0461 (3)	
H7A	0.3928	0.2151	0.5737	0.055*	
C8	0.53032 (12)	0.22258 (9)	0.48457 (16)	0.0525 (4)	
H8A	0.5274	0.1699	0.4453	0.063*	
C9	0.61650 (13)	0.27393 (10)	0.46379 (17)	0.0576 (4)	
H9A	0.6700	0.2555	0.4095	0.069*	
C10	0.62263 (11)	0.35095 (10)	0.52281 (17)	0.0524 (4)	
H10A	0.6801	0.3846	0.5080	0.063*	
C11	0.54257 (10)	0.37989 (9)	0.60603 (14)	0.0425 (3)	
C12	0.54691 (10)	0.46009 (9)	0.67061 (15)	0.0442 (3)	
C13	0.46888 (11)	0.48548 (8)	0.75189 (14)	0.0441 (3)	
H13A	0.4737	0.5370	0.7953	0.053*	
C14	0.22577 (11)	0.52880 (8)	0.77811 (14)	0.0437 (3)	
C15	0.17007 (12)	0.50829 (10)	0.65164 (16)	0.0526 (4)	
H15A	0.1735	0.4547	0.6170	0.063*	
C16	0.10939 (14)	0.56660 (12)	0.57628 (19)	0.0671 (5)	
H16A	0.0721	0.5526	0.4913	0.081*	
C17	0.10514 (15)	0.64454 (13)	0.6283 (2)	0.0727 (6)	
C18	0.15756 (18)	0.66735 (11)	0.7526 (2)	0.0810 (6)	
H18A	0.1526	0.7210	0.7866	0.097*	
C19	0.21853 (14)	0.60881 (10)	0.82764 (19)	0.0622 (4)	
H19A	0.2552	0.6235	0.9127	0.075*	
C20	0.64028 (13)	0.58658 (9)	0.69998 (18)	0.0575 (4)	
H20A	0.7007	0.6141	0.6667	0.086*	
H20B	0.6474	0.5836	0.8016	0.086*	
H20C	0.5774	0.6168	0.6702	0.086*	
C21	0.16104 (11)	0.40092 (9)	1.01911 (14)	0.0442 (3)	
C22	0.09169 (15)	0.49981 (11)	1.17445 (17)	0.0610 (4)	
H22A	0.1145	0.4732	1.2635	0.073*	
H22C	0.0203	0.4818	1.1458	0.073*	
C23	0.09548 (19)	0.59157 (12)	1.19019 (19)	0.0759 (5)	
H23A	0.0444	0.6088	1.2541	0.114*	
H23D	0.0797	0.6169	1.0993	0.114*	
H23C	0.1648	0.6080	1.2273	0.114*	
H2N1	0.1556 (15)	0.2184 (12)	0.773 (2)	0.068 (5)*	
H1N1	0.1147 (16)	0.2602 (11)	0.906 (2)	0.072 (6)*	

### Atomic displacement parameters (Å^2^)

**Table:**

	*U*^11^	*U*^22^	*U*^33^	*U*^12^	*U*^13^	*U*^23^
F1	0.1074 (10)	0.1103 (10)	0.1298 (11)	0.0645 (8)	0.0372 (8)	0.0582 (9)
O1	0.0469 (5)	0.0443 (5)	0.0570 (6)	−0.0104 (4)	0.0167 (4)	−0.0092 (4)
O2	0.0475 (6)	0.0507 (6)	0.0788 (7)	−0.0143 (5)	0.0166 (5)	−0.0054 (5)
O3	0.0612 (6)	0.0524 (6)	0.0566 (6)	−0.0015 (5)	0.0200 (5)	0.0075 (5)
O4	0.0598 (6)	0.0483 (6)	0.0486 (5)	0.0028 (5)	0.0162 (4)	−0.0032 (4)
N1	0.0545 (7)	0.0450 (7)	0.0694 (8)	−0.0103 (6)	0.0231 (6)	−0.0064 (6)
C1	0.0402 (7)	0.0416 (7)	0.0478 (7)	−0.0003 (6)	0.0087 (5)	0.0035 (6)
C2	0.0423 (7)	0.0396 (7)	0.0434 (7)	0.0014 (5)	0.0067 (5)	0.0027 (5)
C3	0.0433 (7)	0.0377 (7)	0.0434 (7)	−0.0023 (5)	0.0058 (5)	−0.0030 (5)
C4	0.0392 (6)	0.0393 (7)	0.0441 (7)	−0.0001 (5)	0.0048 (5)	0.0020 (5)
C5	0.0372 (6)	0.0391 (7)	0.0454 (7)	−0.0051 (5)	0.0064 (5)	0.0029 (5)
C6	0.0416 (7)	0.0389 (7)	0.0411 (6)	0.0001 (5)	0.0039 (5)	0.0025 (5)
C7	0.0495 (8)	0.0424 (7)	0.0469 (7)	−0.0050 (6)	0.0066 (6)	−0.0001 (6)
C8	0.0584 (9)	0.0456 (8)	0.0543 (8)	0.0028 (7)	0.0090 (7)	−0.0080 (6)
C9	0.0524 (8)	0.0589 (9)	0.0635 (9)	0.0049 (7)	0.0184 (7)	−0.0057 (7)
C10	0.0439 (7)	0.0524 (9)	0.0623 (9)	−0.0034 (6)	0.0129 (6)	−0.0003 (7)
C11	0.0404 (7)	0.0426 (7)	0.0448 (7)	−0.0016 (6)	0.0054 (5)	0.0022 (5)
C12	0.0386 (7)	0.0424 (7)	0.0518 (7)	−0.0062 (6)	0.0044 (6)	0.0035 (6)
C13	0.0448 (7)	0.0370 (7)	0.0506 (7)	−0.0036 (6)	0.0037 (6)	−0.0016 (5)
C14	0.0434 (7)	0.0379 (7)	0.0515 (7)	−0.0014 (6)	0.0147 (6)	0.0025 (5)
C15	0.0534 (8)	0.0487 (8)	0.0560 (8)	0.0019 (7)	0.0069 (7)	0.0054 (6)
C16	0.0567 (9)	0.0809 (13)	0.0644 (10)	0.0084 (9)	0.0101 (8)	0.0218 (9)
C17	0.0642 (10)	0.0712 (12)	0.0866 (13)	0.0283 (9)	0.0316 (10)	0.0321 (10)
C18	0.1016 (16)	0.0440 (10)	0.1021 (15)	0.0229 (10)	0.0396 (13)	0.0101 (9)
C19	0.0748 (11)	0.0420 (8)	0.0715 (10)	0.0022 (7)	0.0179 (8)	−0.0034 (7)
C20	0.0520 (8)	0.0460 (8)	0.0738 (10)	−0.0141 (7)	0.0003 (7)	0.0007 (7)
C21	0.0447 (7)	0.0446 (8)	0.0433 (7)	0.0037 (6)	0.0043 (6)	0.0054 (6)
C22	0.0720 (10)	0.0627 (10)	0.0506 (8)	0.0126 (8)	0.0203 (7)	−0.0010 (7)
C23	0.1027 (15)	0.0662 (11)	0.0607 (10)	0.0248 (10)	0.0188 (9)	−0.0052 (8)

### Geometric parameters (Å, º)

**Table:**

F1—C17	1.364 (2)	C9—C10	1.366 (2)
O1—C1	1.3606 (16)	C9—H9A	0.9300
O1—C5	1.3942 (15)	C10—C11	1.4106 (19)
O2—C12	1.3619 (16)	C10—H10A	0.9300
O2—C20	1.4188 (18)	C11—C12	1.4346 (19)
O3—C21	1.2254 (17)	C12—C13	1.3615 (19)
O4—C21	1.3475 (17)	C13—H13A	0.9300
O4—C22	1.4432 (17)	C14—C19	1.383 (2)
N1—C1	1.3367 (18)	C14—C15	1.383 (2)
N1—H2N1	0.89 (2)	C15—C16	1.382 (2)
N1—H1N1	0.88 (2)	C15—H15A	0.9300
C1—C2	1.3668 (19)	C16—C17	1.357 (3)
C2—C21	1.4431 (18)	C16—H16A	0.9300
C2—C3	1.5156 (18)	C17—C18	1.358 (3)
C3—C4	1.5109 (18)	C18—C19	1.385 (3)
C3—C14	1.5236 (19)	C18—H18A	0.9300
C3—H3A	0.9800	C19—H19A	0.9300
C4—C5	1.3568 (19)	C20—H20A	0.9600
C4—C13	1.4173 (18)	C20—H20B	0.9600
C5—C6	1.4191 (18)	C20—H20C	0.9600
C6—C7	1.4137 (18)	C22—C23	1.494 (2)
C6—C11	1.4151 (18)	C22—H22A	0.9700
C7—C8	1.365 (2)	C22—H22C	0.9700
C7—H7A	0.9300	C23—H23A	0.9600
C8—C9	1.399 (2)	C23—H23D	0.9600
C8—H8A	0.9300	C23—H23C	0.9600
			
C1—O1—C5	118.20 (10)	O2—C12—C11	114.43 (12)
C12—O2—C20	117.07 (12)	C12—C13—C4	120.84 (13)
C21—O4—C22	117.42 (12)	C12—C13—H13A	119.6
C1—N1—H2N1	117.5 (12)	C4—C13—H13A	119.6
C1—N1—H1N1	115.7 (13)	C19—C14—C15	118.37 (14)
H2N1—N1—H1N1	121.7 (17)	C19—C14—C3	121.43 (14)
N1—C1—O1	110.17 (12)	C15—C14—C3	120.20 (13)
N1—C1—C2	127.59 (13)	C16—C15—C14	120.71 (16)
O1—C1—C2	122.24 (12)	C16—C15—H15A	119.6
C1—C2—C21	119.41 (12)	C14—C15—H15A	119.6
C1—C2—C3	120.70 (11)	C17—C16—C15	118.97 (18)
C21—C2—C3	119.47 (12)	C17—C16—H16A	120.5
C4—C3—C2	109.69 (11)	C15—C16—H16A	120.5
C4—C3—C14	110.40 (11)	C16—C17—C18	122.44 (16)
C2—C3—C14	112.54 (11)	C16—C17—F1	118.6 (2)
C4—C3—H3A	108.0	C18—C17—F1	118.98 (19)
C2—C3—H3A	108.0	C17—C18—C19	118.42 (17)
C14—C3—H3A	108.0	C17—C18—H18A	120.8
C5—C4—C13	119.14 (12)	C19—C18—H18A	120.8
C5—C4—C3	120.69 (12)	C14—C19—C18	121.09 (18)
C13—C4—C3	120.16 (12)	C14—C19—H19A	119.5
C4—C5—O1	122.35 (11)	C18—C19—H19A	119.5
C4—C5—C6	122.52 (12)	O2—C20—H20A	109.5
O1—C5—C6	115.12 (11)	O2—C20—H20B	109.5
C7—C6—C11	118.93 (12)	H20A—C20—H20B	109.5
C7—C6—C5	123.01 (12)	O2—C20—H20C	109.5
C11—C6—C5	118.06 (12)	H20A—C20—H20C	109.5
C8—C7—C6	120.59 (13)	H20B—C20—H20C	109.5
C8—C7—H7A	119.7	O3—C21—O4	121.78 (12)
C6—C7—H7A	119.7	O3—C21—C2	127.02 (13)
C7—C8—C9	120.42 (14)	O4—C21—C2	111.19 (12)
C7—C8—H8A	119.8	O4—C22—C23	106.36 (14)
C9—C8—H8A	119.8	O4—C22—H22A	110.5
C10—C9—C8	120.42 (14)	C23—C22—H22A	110.5
C10—C9—H9A	119.8	O4—C22—H22C	110.5
C8—C9—H9A	119.8	C23—C22—H22C	110.5
C9—C10—C11	120.66 (14)	H22A—C22—H22C	108.6
C9—C10—H10A	119.7	C22—C23—H23A	109.5
C11—C10—H10A	119.7	C22—C23—H23D	109.5
C10—C11—C6	118.95 (13)	H23A—C23—H23D	109.5
C10—C11—C12	122.14 (13)	C22—C23—H23C	109.5
C6—C11—C12	118.90 (12)	H23A—C23—H23C	109.5
C13—C12—O2	125.08 (13)	H23D—C23—H23C	109.5
C13—C12—C11	120.49 (12)		
			
C5—O1—C1—N1	−166.73 (12)	C7—C6—C11—C12	−179.64 (12)
C5—O1—C1—C2	13.33 (19)	C5—C6—C11—C12	0.08 (19)
N1—C1—C2—C21	1.2 (2)	C20—O2—C12—C13	−2.1 (2)
O1—C1—C2—C21	−178.85 (12)	C20—O2—C12—C11	177.94 (12)
N1—C1—C2—C3	−171.35 (14)	C10—C11—C12—C13	−179.08 (13)
O1—C1—C2—C3	8.6 (2)	C6—C11—C12—C13	1.7 (2)
C1—C2—C3—C4	−24.06 (17)	C10—C11—C12—O2	0.9 (2)
C21—C2—C3—C4	163.38 (11)	C6—C11—C12—O2	−178.31 (12)
C1—C2—C3—C14	99.26 (15)	O2—C12—C13—C4	178.23 (13)
C21—C2—C3—C14	−73.30 (15)	C11—C12—C13—C4	−1.8 (2)
C2—C3—C4—C5	20.10 (17)	C5—C4—C13—C12	0.0 (2)
C14—C3—C4—C5	−104.48 (14)	C3—C4—C13—C12	−178.74 (12)
C2—C3—C4—C13	−161.16 (12)	C4—C3—C14—C19	−117.90 (14)
C14—C3—C4—C13	74.27 (15)	C2—C3—C14—C19	119.17 (14)
C13—C4—C5—O1	−179.49 (12)	C4—C3—C14—C15	61.86 (16)
C3—C4—C5—O1	−0.7 (2)	C2—C3—C14—C15	−61.06 (16)
C13—C4—C5—C6	1.8 (2)	C19—C14—C15—C16	0.5 (2)
C3—C4—C5—C6	−179.41 (12)	C3—C14—C15—C16	−179.29 (13)
C1—O1—C5—C4	−17.49 (19)	C14—C15—C16—C17	0.0 (2)
C1—O1—C5—C6	161.27 (11)	C15—C16—C17—C18	−0.7 (3)
C4—C5—C6—C7	177.85 (12)	C15—C16—C17—F1	178.41 (15)
O1—C5—C6—C7	−0.91 (18)	C16—C17—C18—C19	0.8 (3)
C4—C5—C6—C11	−1.9 (2)	F1—C17—C18—C19	−178.22 (16)
O1—C5—C6—C11	179.37 (11)	C15—C14—C19—C18	−0.3 (2)
C11—C6—C7—C8	0.2 (2)	C3—C14—C19—C18	179.47 (15)
C5—C6—C7—C8	−179.52 (13)	C17—C18—C19—C14	−0.4 (3)
C6—C7—C8—C9	−1.3 (2)	C22—O4—C21—O3	−5.2 (2)
C7—C8—C9—C10	1.1 (2)	C22—O4—C21—C2	175.45 (12)
C8—C9—C10—C11	0.3 (2)	C1—C2—C21—O3	10.6 (2)
C9—C10—C11—C6	−1.3 (2)	C3—C2—C21—O3	−176.74 (13)
C9—C10—C11—C12	179.44 (14)	C1—C2—C21—O4	−170.03 (12)
C7—C6—C11—C10	1.11 (19)	C3—C2—C21—O4	2.62 (17)
C5—C6—C11—C10	−179.17 (12)	C21—O4—C22—C23	−170.70 (14)

### Hydrogen-bond geometry (Å, º)

*Cg*1, *Cg*2 and *Cg*3 are the centroids of C4–C6/C11–C13, C14–C19 and C6–C11 rings, respectively.

**Table:**

*D*—H···*A*	*D*—H	H···*A*	*D*···*A*	*D*—H···*A*
N1—H2*N*1···O3^i^	0.89 (2)	2.23 (2)	3.0969 (19)	165.8 (17)
N1—H1*N*1···O3	0.89 (2)	2.111 (18)	2.7570 (18)	129.1 (16)
N1—H1*N*1···F1^ii^	0.89 (2)	2.32 (2)	3.034 (2)	137.6 (16)
C8—H8*A*···*Cg*1^iii^	0.93	2.81	3.5633 (16)	139
C10—H10*A*···*Cg*2^iv^	0.93	2.94	3.7003 (17)	140
C20—H20*C*···*Cg*3^iv^	0.96	2.74	3.5896 (17)	148

Symmetry codes: (i) *x*, −*y*+1/2, *z*−1/2; (ii) −*x*, *y*−1/2, −*z*+3/2; (iii) *x*, −*y*−1/2, *z*−3/2; (iv) −*x*+1, −*y*+1, −*z*+1.
